# Review of therapies for intermediate and advanced stage hepatocellular carcinoma, not suitable for curative therapies: a rapidly changing landscape

**DOI:** 10.20517/2394-5079.2018.113

**Published:** 2019-01-24

**Authors:** Brian I. Carr

**Affiliations:** Liver Transplantation Institute, Inonu University, Malatya, Turkey and Izmir Biomedicine and Genome Center, Dokuz Eylul University, Izmir 35340, Turkey.

**Keywords:** Hepatocellular carcinoma, advanced, kinase inhibitors, immune checkpoint inhibitors, transarterial chemoembolization, selective internal radiation therapy

## Abstract

Recent clinical trials and new agents have permitted greater clarity in the choice of effective agents for that majority of patients with hepatocellular carcinoma who have advanced disease at diagnosis and thus cannot be offered potentially curative resection, ablation or liver transplantation. The main treatment for these patients remains chemoembolization, although evidence for selective internal radiation therapy (SIRT) with SIR-Spheres or Theraphere, is beginning to suggest that the results with this may be comparable with less toxicity. Patients who have failed chemoembolization or SIRT or have metastatic disease at presentation are suitable for the multikinase inhibitor sorafenib (nexavar) or newly-approved lenvatinib (lenvima) as first line therapies. The choice between which of them to use first is not currently clear. Patients who have failed sorafenib can be offered a choice of FDA-approved regorafenib (stivarga) or immune checkpoint inhibitor nivolumab (opdivo) as second line agents. For that considerable percent of patients presenting with macroscopic portal vein thrombosis, the choice appears to be between multikinase inhibitor or SIRT, given the potential toxicity of chemoembolization in this setting. However, considering the potency of both nivolumab and regorafenib and the pipeline of new agents such as atezolizumab (tecentriq) in current clinical trials, including new immune checkpoint inhibitors, this landscape may change within a couple of years, especially if new evidence arises for the superior effectiveness of combinations of any of these agents over single agents.

## INTRODUCTION

The prognosis of hepatocellular carcinoma (HCC) depends on multiple factors, hence the large number of staging systems. In particular, it depends on the size and location within the liver of the HCC and the number of HCC nodules, the presence and degree of portal venous invasion, the presence or absence of distant metastases, as well as the degree of liver damage (Child-Pugh class)^[1]^. Patients with a single tumor nodule of < 2 cm have the best prognosis and larger size and number of nodules have worse prognosis^[2]^. T1 lesions are single < 2 cm lesions without portal vein thrombosis (PVT). T2 lesions are > 2 cm to 5 cm, single or multiple, as well as single lesions > 2 cm with vascular invasion. T3 lesions are multiple, with at least one being > 5 cm. The best survival outcomes occur after treatment with ablation, resection or transplantation, in patients having Barcelona Clinic Liver Cancer staging system (BCLC)^[3]^ 0 stage (single < 2 cm) or early stage A (1–3 nodules, any < 3 cm with good liver function Child-Pugh class A or early class B cirrhosis). All other patients, who have BCLC stages B (multinodular of any size) or stage C (presence of PVT, lymph nodes or metastases), cannot be offered therapies with curative intent, and constitute the majority of HCC patients who are diagnosed in the absence of a surveillance program and whose treatment is the subject of this review. This constituted at least 65% of newly diagnosed HCC patients in our large series^[4]^ [Table 1].

The main treatment modality for these BCLC intermediate stage B patients has been for many years chemoembolization [transarterial chemoembolization (TACE)]. More recently, selective internal radiation therapy (SIRT) or transarterial radioembolization (TARE) has been increasingly seen as a promising treatment approach in this setting, in many institutions. For stage C patients having either or both PVT or metastases, systemic therapy is widely used, involving multikinase inhibitors such as sorafenib, although immune checkpoint inhibitors and newly-approved multikinase inhibitors are changing that landscape. Furthermore, in addition to sorafenib, radioembolization is increasingly considered as both useful and safe (unlike much TACE) in the presence of branch PVT. This review summarizes each of the major treatment modalities for patients who are not suitable for treatments with curative intent and then summarizes current clinical practice and finally evaluates some likely future directions in this rapidly moving field.

## NON-SURGICAL TREATMENT MODALITIES

### Current first line therapies

#### Chemoembolization or TACE

Several reviews have been published on the chemotherapy drugs and types of embolization particles that have been used for chemoembolization or TACE^[4,5]^. Objective partial responses have been reported in 30%−60% of patients^[4]^, and an increase in survival was initially reported in 2 randomized placebo-controlled trials, using doxorubicin or cisplatin, respectively^[6–8]^. Due to its relative safety, especially in patients with Child-Pugh A and many with Child-Pugh B cirrhosis and tumors of almost any size and number, it has been a standard of therapy for non surgical and non metastatic HCC for several decades. A wide range of chemotherapeutic agents have been used, but there has not been an analysis of which agents, or combination of agents, nor of which of multiple embolization particle types might be optimal, although doxorubicin, cisplatin or mitomycin C, often mixed with lipiodol, are most commonly used^[9]^, or with defined size embolization particles. Recently, drug-eluting beads have become popular, but their superiority for survival to plain and cheaper particles has been disputed^[10]^, although they may be safer. TACE has been combined with radiofrequency invasion for enhanced results^[11]^ and has also been used as bridging therapy to transplant^[12]^. Current trials are in progress to assess improved efficacy of TACE when combined with multikinase inhibitors^[13,14]^ or immune checkpoint inhibitors. TACE has been considered the standard therapy for non surgically treatable HCC^[15]^, with SIRT being also widely adopted as an alternative standard.

#### SIRT

TARE with SIR-Spheres or Transarterial radiotherapy with Therasphere [Table 1].

Yttrium-90 or Y^90^ SIRT has gained increased popularity in recent years as a safer alternative to TACE, especially in the setting of PVT. Two non-identical products [Table 1] are available, namely Therasphere and SIR-Spheres. Therasphere contains glass as the carrier and is much more radioactive, but almost not embolic and is FDA-approved for HCC therapy under a humanitarian device exemption (requires individual institutional review board approval). SIR-Spheres are made of resin carrier and are by contrast much less radioactive per dose, but have many more particles per dose and are thus embolic (hence radioembolization). Neither agent seems to induce much post-embolization syndrome, unlike TACE. Therasphere is thus really a pure internal radiation treatment and not radioembolization. There have been few convincing randomized trials with either agent for HCC survival, either against each other (although they are thought to have similar results) or against TACE^[16]^. However, several reports provide evidence for their effectiveness and safety^[16–19]^. Unlike TACE, these radioactive agents need to be received by the institution and handled by radiation safety staff and appropriately monitored. Thus, SIRT therapy requires a special team, including a radiation pharmacy, radiation safety officer, nuclear medicine physician, as well as the interventional radiologist. Unlike for TACE, SIRT patients require a pre-treatment angiogram together with a Technetium ^99m^Tc macro aggregated albumin (^99m^Tc-MAA) scan to measure any significant lung shunting. More than 20% lung shunt normally excludes SIRT, as does aberrant gastric or other feeder arteries than cannot be occluded, to prevent gastrointestinal radiation toxicity.

The most remarkable benefit of SIRT is its safety in treating that 30%−40% of HCC patients that have PVT^[20–22]^. However, overall survival (OS) did not differ significantly when SIRT was compared to sorafenib in a phase III trial^[23]^. Nevertheless, the combination of SIRT with sorafenib was associated in one study with enhanced toxicity^[24]^. In the SORAMIC randomized phase II trial, the addition of SIRT (SIR-Spheres) to sorafenib did not add to survival compared with sorafenib alone. When TACE and SIRT were directly compared, they were similar in safety, tumor responses and survival^[25,26]^. Studies are in progress on the uses of SIRT in adjuvant and neo-adjuvant therapy for surgery of HCC, as well as in combinations with several newer therapies.

#### Sorafenib (nexavar)

Sorafenib is a multikinase inhibitor that is antiangiogenic, inhibits HCC cell growth and induces apoptosis. It is thought to target the Ras/Raf/methyl ethyl ketone (MEK)/extracellular signal-regulated kinase signaling pathway via the vascular endothelial growth factor (VEGFR) and platelet-derived growth factor receptor (PDGFR). For the last 10 years it has been the choice for first line of therapy for patients with HCC metastases, PVT, or those who have failed TACE or SIRT, based on a multi-center, double-blind, placebo-controlled phase III SHARP trial, which reported a 2.8 months increase in median OS with sorafenib (10.7 months) compared with placebo (7.9 months) [hazard ratio (HR) in sorafenib group, 0.69; 95% confidence interval (CI) 0.55–0.87; *P* < 0.007]^[27]^. However, in a similarly designed phase III trial from Asia, results were much worse, with a median OS of 6.5 months (95% CI 5.56–7.56) in patients treated with sorafenib, compared with 4.2 months (3.75–5.46) in those who received placebo (HR 0.68; 95% CI 0.50–0.93; *P* = 0.014)^[28]^. The reasons that the OS from Asia after sorafenib treatment was worse than the OS on placebo in the European study are not clear, but point to the need for caution in comparing results of therapies in different ethnic groups, or in patients with differing severity of tumor or cirrhosis. In addition to a significant but only modest increase in survival in the sorafenib groups compared to placebo controls, the objective response rates of < 2.0% were also very low. However, toxicities have been considerable, with many patients requiring dose reduction, variable drug “holiday” or drug discontinuation. Toxicities include hand-foot syndrome, rash, diarrhea and fatigue, most commonly, but also hypertension, nausea and leukopenia^[29]^.

Several large phase III trials comparing sorafenib with newer agents have failed to successfully meet their planned end-points, including trials of brivanib, linifanib, sunitinib. A randomized phase II trial with sorafenib *vs.* erlotinib plus bevacizumab likewise failed to show superiority for the comparison arm with respect to sorafenib. The onlyrecent exception thus far, is the recently FDA-approved lenvatinib (below) phase III trial.

Several attempts to improve on sorafenib therapy by combining it with other agents or with TACE or SIRT, have been recently made. However, results have so far been minor at best^[30,31]^. Sorafenib was also evaluated as an adjuvant therapy to resection in the STORM trial, but also without added benefit to surgery alone^[32]^.

#### Lenvatinib (lenvima)

FDA has just (Aug 2018) approved lenvatinib for first line therapy of advanced or metastatic HCC, based on a randomized controlled phase III REFLECT trial, comparing lenvatinib 8 or 12 mg daily with sorafenib 400 mg twice daily^[33]^. Median OS was 13.6 months for lenvatinib and 12.3 months for sorafenib. The trial demonstrated that lenvatinib was noninferior (but not statistically superior) to sorafenib for OS, which was the primary endpoint (HR 0.92; 95% CI 0.79–1.06). The overall response rate was higher for lenvatinib than for sorafenib (41% *vs.* 12% per modified RECIST and 19% *vs.* 7% per RECIST 1.1). Patients with main trunk PVT were excluded from this trial. The commonest toxicities in the lenvatinib-treated patients (≥ 20%) were hypertension, fatigue, diarrhea, decreased appetite, arthralgia/myalgia, decreased weight, abdominal pain and palmar-plantar erythrodysaesthesia. It is a multi-tyrosine kinase inhibitor of VEGFR1–3, FGFR 1–4, rearranged during transfection (RET), receptor tyrosine kinase (KIT, also called CD117 and stem cell factor receptor) and PDGFR.

Thus, current first-line therapies for previously untreated HCC, include TACE, SIRT, sorafenib and lenvatinib [Table 2]. The initial choice has been conventional chemoembolization (TACE) or more recently SIRT, especially in the presence of PVT and excellent liver function. However, in the presence of 5 or more lesions or bilobar lesions, it is reasonable to consider Sorafenib or Lenvatinib as initial therapy, especially in the presence of serum bilirubin levels > 2.5 mg/dL, in light of the known hepatotoxicity of both TACE and SIRT.

### Current second line therapies

#### Regorafenib (stivarga)

Regorafenib is a multi-kinase inhibitor of VEGFR1–3, tyrosine kinase with immunoglobulin-like and EGF-like domains 2-unlike sorafenib, PDGFRβ, FGFR, c-KIT (stronger than sorafenib), RET, BRAF, BRAFV600 and RAF-1. It is the first agent to provide survival benefit in the second line, after failure of sorafenib and has recently been FDA-approved as a second line therapy. The phase III RESORCE study^[34]^ was for HCC patients who had progressed on sorafenib, but not failed due to toxicity, and it improved OS with a HR of 0.63 (*P* < 0.0001); the median OS was 10.6 months for regorafenib *vs.* 7.8 months for placebo and the disease control rate was 65.2% *vs.* 36.1% (*P* < 0.001). Regorafenib was administered at 160 mg daily for 3 weeks, with a subsequent rest week. The commonest grade 3 or 4 treatment-emergent events were 15% hypertension in the regorafenib group *vs.* 5% in the placebo group, 13% hand-foot skin reaction/palmar-plantar erythrodysaesthesia for regorafenib *vs.* 1% in the placebo group, 9% fatigue for regorafenib *vs.* 5% in the placebo group, with 3% diarrhea for regorafenib vs. none for placebo. Thus, these data differ from the sorafenib SHARP trial results in which few patients had objective responses, suggesting that regorafenib (fluoro-sorafenib) is a more potent agent than sorafenib. Toxicities were similar for regorafenib and sorafenib, with fatigue, hypertension, hand-foot syndrome, slight elevation of transaminases and bilirubin occurring after both drug treatments. The recommended regorafenib dose is 160 mg per day. The RESORCE trial showed that it is possible to dose-reduce regorafenib and still obtain antitumor effects. Given the remarkable structural similarity between sorafenib and regorafenib - one fluorine atom difference - it is surprising that results for regorafenib were so positive in proven sorafenib-resistant patients. Both sorafenib and regorafenib inhibit the insulin-like growth factor-1 (IGF-1) mediated growth pathway, and their actions *in vitro* are both blocked by IGF-1. By contrast, their actions are augmented by IGF-1 receptor inhibition^[35]^ suggesting future directions for enhancing their effects.

#### Nivolumab (opdivo)

FDA approved nivolumab, a programmed death receptor 1 (PD-1) immune checkpoint inhibitor, as second line therapy for HCC patients who had failed prior sorafenib due to disease progression or sorafenib intolerance, after tumor response and durability of those responses of the single arm phase Ib/II CheckMate-040 trial^[36]^. Results showed that 22 or 14% of 154 patients responded, regardless of their programmed death receptor ligand 1 (PD-L1) status. Three of these patients had complete responses, with 91% of patients having responses lasting 6 months and 55% of patients having responses for more than a year. Median duration of response was 16.6 months, with a rapid median onset of response at 2.8 months. The 12 months OS rate was 59.9% and the median OS was 16.7 months. Serious adverse events occurred in 49% of patients and included pyrexia, ascites, back pains and abdominal pains and general deterioration. Commonest toxicities were 38% of patients had fatigue, 36% musculoskeletal pain, 34% abdominal pain, 27% pruritus, 27% diarrhea, 26% rash and 23% cough. Thus, the toxicity profile is significant and somewhat different from the multikinase inhibitors. The drug can cause immune-mediated colitis, hepatitis, pneumonitis and endocrinopathies. The toxicity results of long-duration therapy are unknown, but may be of concern. On the positive side, the mechanisms of this class of drugs are so different from TACE, SIRT and other multikinase inhibitors, that they will be very attractive candidates for future drug combination trials. A phase III comparison of nivolumab *vs.* sorafenib is ongoing.

Two new agents that have met their end-points in phase III trials in the second-line post sorafenib setting. Ramucirumab (cyramza) is awaiting FDA evaluation and cabozantinib (cabometyx) in the Celestial trial has just been FDA approved.

#### Cabozantinib (cabometyx)

Cabozantinib is a tyrosine kinase inhibitor, with targets including VEGF receptors 1, 2, and 3, MET, and AXL, which are implicated in the HCC growth and sorafenib resistance. A phase III placebo controlled trial was reported^[37]^, showing that in sorafenib resistant patients, cabozantinib treatment resulted in longer OS than for placebo patients. Median OS was 10.2 months with cabozantinib and 8.0 months with placebo (HR for death, 0.76; 95% CI 0.63–0.92; *P* = 0.005), and the objective response rates were 4% for cabozantinib, but less than 0.4% for placebo, respectively (*P* = 0.009). 16% of patients discontinued cabozantinib due to treatment-related adverse events (palmar-plantar erythrodysesthesia, hypertension, fatigue, diarrhea and increased aspartate aminotransferase), compared to 3% of patients on placebo. Given that the phase III trial also included patients receiving cabozantinib as third line therapy, this opens the possibility for the potential for third line therapies in patients with resistant HCC or who are intolerant to other therapies.

#### Ramucirumab (cyramza)

Ramucirumab is an anti-angiogenic VEGFR-2 antagonist that binds and blocks VEGF-A, VEGF-C and VEGF-D. In a phase III placebo-controlled trial (REACH) in second line on sorafenib failure patients, no significant survival differences were found between ramucirumab and placebo. However, meaningful improvement was observed in a patient subgroup with baseline alpha-fetoprotein (AFP) ≥ 400 ng/mL, HR = 0.67, *P* = 0.006; median OS 7.8 months for ramucirumab *vs.* 4.2 months for placebo controls. Therefore, a subsequent phase III randomized trial was performed (REACH-2), in a biomarker-selected HCC patients, having AFP levels of > 400 ng/mL^[38]^. In patients with baseline AFP ≥ 400 ng/mL, a significant survival benefit was found in patients treated with ramucirumab compared with placebo and was coupled with a trend in patient-focused outcome benefits. The only grade 3 toxicity was hypertension and hyponatremia in > 5% of the patients. In a Japanese sub-analysis^[39]^, the median OS was 12.9 months for the ramucirumab arm (*n* = 45) and 8.0 months for the placebo arm (*n* = 48) (HR 0.621; 95% CI 0.391–0.986; *P* = 0.0416). In patients with a baseline AFP level of 400 ng/mL or greater, the median OS was 12.9 months for the ramucirumab arm (*n* = 20) and 4.3 months for the placebo arm (*n* = 22) (HR 0.464; 95% CI 0.232–0.926); *P* = 0.0263). Objective response rates were 11% for the ramucirumab arm and 2% for the placebo arm (*P* = 0.0817). Ramucirumab is currently being considered for approval by the FDA.

Thus, 3 agents are currently FDA-approved for second line therapy, namely regorafenib, nivolumab and cabozantinib. However, in 2019 ramucirumab may also be approved in this same setting. How does one choose the optimal sequence for using these agents? In addition, for liver-only HCC patients who have failed chemoembolization and who have preserved liver function, may also be suitably treated with radioembolization. Given the high response rates for regorafenib, this is an attractive agent for use in this setting, but its use is also associated with considerable toxicities. The RESOURCE trial on which its approval was based, did not include patients who were sorafenib-intolerant in the first line setting. Thus, the use of regorafenib in the second line setting may be limited to a subset of patients. Cabozantinib and ramucirumab are also multikinase inhibitors, with similar toxicities to both sorafenib and regorafenib. Therefore, patients whose tumors have failed chemoembolization and/or radioembolization might be most suitably offered nivolumab at the time of writing, due to its different toxicities and even higher responses. New approvals are likely however, for other immune checkpoint inhibitors and/or their combinations with other agents and these recommendations will then need to be reconsidered.

## EXTRA-HEPATIC METASTASIS AND PVT

Metastasis is the single most important cause of morbidity and mortality in most solid adult tumors. HCC may be an exception, as patients usually die of their liver failure, either from tumor growth and parenchymal liver destruction, or from the underlying and liver disease that caused the HCC to arise, regardless of the presence or absence of metastasis. HCC with extra-hepatic metastasis may even constitute a distinct HCC subset, and is associated with less cirrhosis than other HCC^[40]^. While systemic therapy is mainly chosen in this circumstance^[41]^, an argument can also be made to initially treat the main disease in the liver. Regardless, several studies with systemic chemotherapy or multikinase therapy have shown no survival benefit in this situation.

Macroscopic PVT (visible on MRI or CT scan) is thought to be present in over 30% of HCC patients and is likely the single worst prognostic factor. In addition to being an important portal for metastases (tumor cells are already in the portal vein), the presence of main stem or major branch PVT impacts the ability to perform liver transplant (high recurrence rates), resection (high recurrence rates and technical surgical difficulties); it is also associated with worse liver function. Many studies have thus focused on the treatment of HCC patients with PVT^[41]^, as well as on treatment of the PVT itself^[41,42]^. Treatments include selective TACE, SIRT^[20–22,43]^, sorafenib and 3-dimensional conformal radiotherapy. However, this is a heterogeneous group of patients^[44,45]^. One consensus suggests hepatic resection when technically feasible for longest survival, otherwise TACE for unresectable patients, followed by external beam radiation^[46]^. Depending on the extent of the PVT, enhanced survival has been reported in a large series for hepatectomy, TACE, TACE plus sorafenib or TACE plus radiotherapy^[47]^. There is currently no standard for therapy for PVT.

## LIKELY PRACTICE SCENARIOS [Table 2]

FDA has approved both sorafenib and lenvatinib as first line therapies. If patients tolerate sorafenib well, then FDA-approved regorafenib or nivolumab will be good second line options. If patients did not tolerate sorafenib, then FDA-approved nivolumab might be an excellent second line option, due to its different mechanisms than sorafenib. But so could ramucirumab, should it get approved by FDA in the second line setting. Furthermore, ramucirumab appears to be attractive for patients in the second line setting with elevated AFP levels.

## NEW AGENTS

A variety of new agents are in current clinical trials and will likely change the clinical landscape again in another 2–5 years. These include particularly a variety of agents inhibiting the immune checkpoint proteins PD-1, PD-L1 and cytotoxic T lymphocyte antigen 4, as well as epigenetic control mechanisms. In addition to further opdivo studies, other agents being tested include ipilumimab, prembrolizumab and durvalumab and tremelimumab, amongst others. Agents against various growth factor targets such as FGF/FGFR (fibroblast growth factor and its receptor, such as BLU 554 or dovatinib) and growth pathways (MEK, signal transducer and activator of transcription 3, AKT-also called protein kinase B), apoptosis induction, epithelial to mesenchymal modulation, and cytolytic viruses, are currently under way. Furthermore, the clinical availability of curative (HCV) or highly effective (HBV) antivirals that are the ultimate cause of HCC and hoped-for contributors to the amelioration of HCC aggressiveness. In this context, the role of inflammatory micro-environment and anti-inflammatory agents in the development of HCC and its modulation, is drawing increased interest.

## WHERE ARE WE HEADING?

The standard of care for patients with advanced, non-curative and non metastastic HCC remains TACE or more recently, TARE [National Comprehensive Cancer Network (NCCN) guidelines version 5.2018, HCC; NCCN.org]. Chemoembolization is associated with 30%−60% objective response rates in various trials and has some minor survival advantage^[6,7]^. Sorafenib has minor response rates, can be given orally and has a proven, but small survival advantage, through quite different mechanisms and different toxicity profiles than for either TACE or TARE. Therefore, it will be rational to evaluate combinations of chemoembolization or SIRT with sorafenib^[48,49]^ or the more potent regorafenib or any other multikinase inhibitor. Several trials are under way.

The same reasoning of different mechanisms, applies to combinations of immune checkpoint inhibitor with either: (a) chemoembolization; (b) SIRT; and (c) multikinase inhibitors. In this regard, the combination of VEGF inhibitor bevacizumab (avastin) plus atezolizumab (tecentriq) has just been given (July 2018) breakthrough therapy designation by FDA, since a phase Ib study presented at American Society of Clinical Oncology (ASCO) 2018 was reported to show objective responses in 32% of patients. More than half of the responders maintained their responses for at least 6 months. A combination trial of nivolumab plus sorafenib (CheckMate-459) for first line therapy is currently in progress. Furthermore, combinations of kinase inhibitors that target different or parallel growth pathways [EGFR, FGFR, hepatocyte growth factor receptor (HGFR)/Met] seems similarly attractive for testing. In addition, it may be that sequencing might show added anti-tumor activity rather than combinations, such as chemoembolization/sorafenib, SIRT/sorafenib, sorafenib/regorafenib, immune checkpoint inhibitor (high responses)/multikinase inhibitor. Thus, the field may look quite differently in 3 years than currently. The role of anti-viral or anti-inflammatory agents (above section) may also turn out to be beneficial in selected patient subsets, with greater inflammatory characteristics^[50]^. Either way, HCC sub-phenotype identification may be important in matching individuals to selected treatments. However, a final word of caution may be useful. A major phase III trial recently failed to meet its end-points, even though patients were selected, based on their tumors having the putative target (Met) for the agent being tested^[51]^. However, given the high responses and their durability for immune checkpoint inhibitors such as Nivolumab, one of them may become a preferred first line therapy, if ongoing clinical trials support this idea.

## Figures and Tables

**Table 1.** Comparison between glass (Therasphere) and resin microspheres (SIR-Spheres)

	Therasphere	SIR-Spheres
Half-life	64.2 h	64.2 h
Material	Glass	Resin
Size	20–30 μm	20–60 μm
Activity per sphere	2500 Bq	50 Bq
Number of sphere	1.2–8 milion	40–80 milion
Embolic effect	Minimal	Moderate

**Table 2.** Current therapies for advanced stage hepatocellular carcinoma patients

First line
A. Chemoembolization or SIRT
B. Sorafenib or Lenvatinib
Second line
A. Regorafenib or Opdivo or Cabozantinib
B. Under FDA review: ramucirumab
Metastasis
Sorafenib or Lenvatinib
PVT
SIRT, Sorafenib, external beam irradiation
Combinations in development
A. Multikinase inhibitors plus chemoembolization or SIRT
B. Immune checkpoint inhibitors plus chemoembolization or SIRT
C. Kinase inhibitors targeting parallel growth pathways
D. Multikinase inhibitors plus immune checkpoint inhibitors

SIRT: selective internal radiation therapy; PVT: portal vein thrombosis; SBRT: stereotactic body radiation therapy
